# Supplementary material for: Inflammatory factors and risk of lung adenocarcinoma: a Mendelian randomization study mediated by blood metabolites
Source: Front Endocrinol (Lausanne). 2024 Aug 27;15:1446863. doi: 10.3389/fendo.2024.1446863 (PMC11384989; doi:10.3389/fendo.2024.1446863)
Supplement: Supplementary file 1 [file DataSheet1.zip › supplementary files/S2/GCST90200637/sensitivity-analysis.pdf]

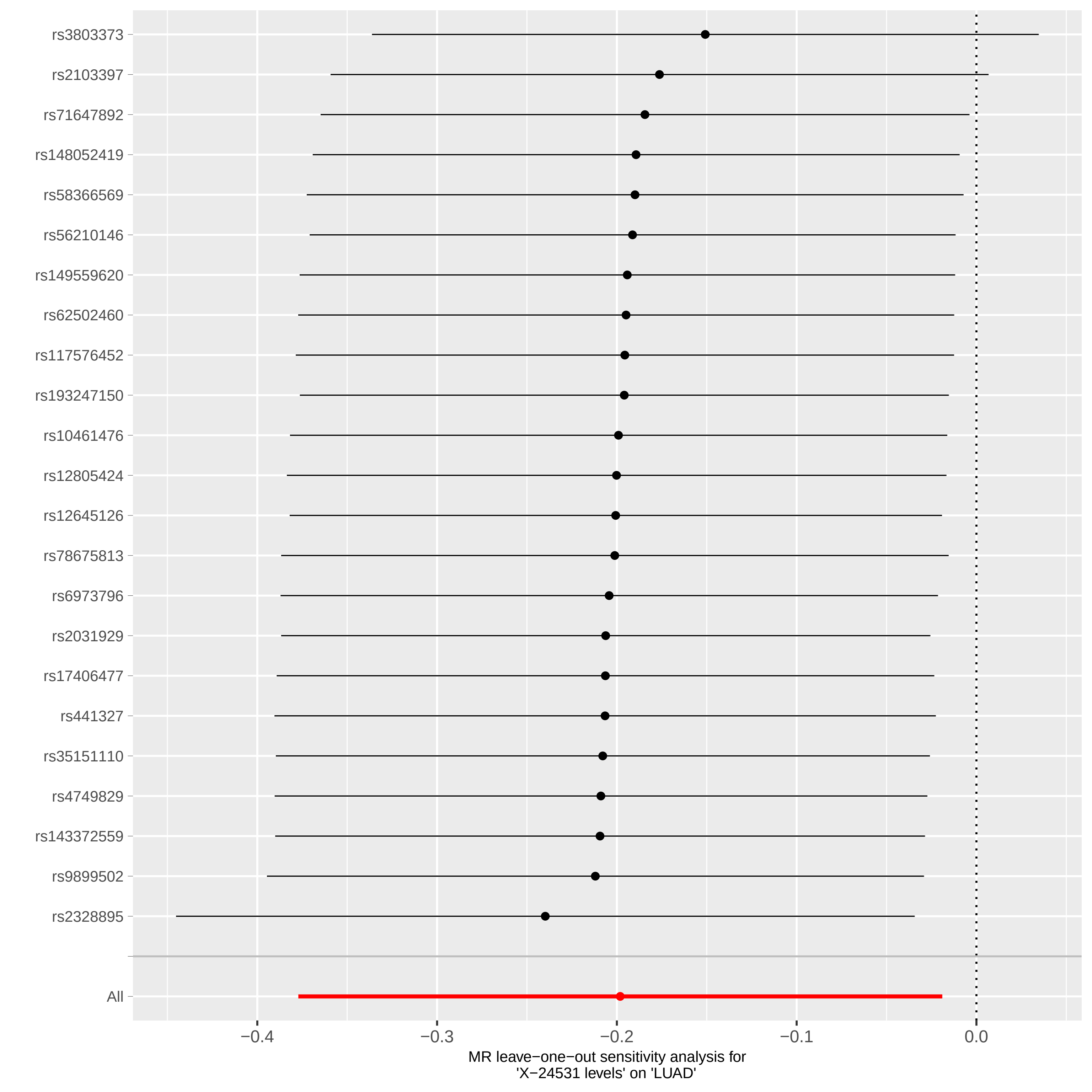
rs3803373
rs2103397
rs71647892
rs148052419
rs58366569
rs56210146
rs149559620
rs62502460
rs117576452
rs193247150
rs10461476
rs12805424
rs12645126
rs78675813
rs6973796
rs2031929
rs17406477
rs441327
rs35151110
rs4749829
rs143372559
rs9899502
rs2328895
All
−0.4
−0.3
−0.2
−0.1
0.0
MR leave−one−out sensitivity analysis for
'X−24531 levels' on 'LUAD'
